# Relationship of Corpus Callosum Integrity with Working Memory, Planning, and Speed of Processing in Patients with First-Episode and Chronic Schizophrenia

**DOI:** 10.3390/jcm10143158

**Published:** 2021-07-17

**Authors:** Ernest Tyburski, Piotr Podwalski, Katarzyna Waszczuk, Katarzyna Rek-Owodziń, Monika Mak, Piotr Plichta, Maksymilian Bielecki, Krzysztof Rudkowski, Michał Szelepajło, Jolanta Kucharska-Mazur, Wojciech Andrusewicz, Błażej Misiak, Magdalena Kerestey, Adrianna Bober, Krzysztof Wietrzyński, Anna Michalczyk, Magdalena Więdłocha, Piotr Marcinowicz, Jerzy Samochowiec, Leszek Sagan

**Affiliations:** 1 Institute of Psychology, SWPS University of Social Sciences and Humanities, 61-719 Poznań, Poland; 2 Department of Psychiatry, Pomeranian Medical University in Szczecin, 71-457 Szczecin, Poland; piotr.podwalski@gmail.com (P.P.); zurawska1989@gmail.com (K.W.); krudkowski@gmail.com (K.R.); michalszelepajlo@wp.pl (M.S.); jola_kucharska@tlen.pl (J.K.-M.); annakarolina6@wp.pl (A.M.); samoj@pum.edu.pl (J.S.); 3 Department of Health Psychology, Pomeranian Medical University in Szczecin, 71-457 Szczecin, Poland; katarzynarek90@gmail.com (K.R.-O.); monika.mak@gmail.com (M.M.); piotrpp119@gmail.com (P.P.); maksbiel@gmail.com (M.B.); krzysztofwietrzynski@gmail.com (K.W.); 4 Department of Neurosurgery, Pomeranian Medical University in Szczecin, 71-252 Szczecin, Poland; wojciech.andrusewicz@gmail.com (W.A.); leszekm.sagan@gmail.com (L.S.); 5 Department of Psychiatry, Division of Consultation Psychiatry and Neuroscience, Wroclaw Medical University, 50-367 Wroclaw, Poland; mblazej@interia.eu; 6 Institute of Psychology, University of Szczecin, 71-017 Szczecin, Poland; magdalena@kerestey.net (M.K.); adrianaweronika@gmail.com (A.B.); 7 Department of Psychiatry, Faculty of Health Sciences, Medical University in Warsaw, 02-091 Warsaw, Poland; magdalena.wiedlocha@wum.edu.pl (M.W.); piotr.marcinowicz@gmail.com (P.M.)

**Keywords:** corpus callosum, working memory, planning, speed of processing, first-episode schizophrenia, chronic schizophrenia, diffusion tensor imaging, dti, fractional anisotropy, mean diffusivity

## Abstract

There is a paucity of reports examining the relationship between the integrity of the corpus callosum (CC) and different aspects of cognitive functioning in patients with first-episode (FES) and chronic schizophrenia (CS) simultaneously; furthermore, what results exist are inconclusive. We used diffusion tensor imaging tractography to investigate differences in integrity in five regions of the CC between FES, CS, and healthy controls (HC). Additionally, we analyzed correlations between these regions’ integrity and working memory, planning, and speed of processing. Eighteen patients with FES, 55 patients with CS, and 30 HC took part in the study. We assessed cognitive functions with four tasks from Measurement and Treatment Research to Improve Cognition in Schizophrenia. Patients with CS showed lower fractional anisotropy (FA) in Region 5 (statistical trend) and higher mean diffusivity (MD) in Regions 4 and 5 than HC, and patients with FES had higher MD in Region 3 (statistical trend) than HC. Both clinical groups performed worse on working memory and speed of processing tasks than HC, and patients with CS scored worse than HC on independent planning, and worse than FES and HC on dependent planning. Moreover, in patients with CS, MD in Region 3 was correlated with verbal working memory. Our results suggest that patients with FES and CS are characterized by impaired integrity of the middle and posterior CC, respectively. We confirmed that both clinical groups have cognitive impairments. Moreover, the integrity of the middle CC may influence planning in patients with CS.

## 1. Introduction

Cognitive dysfunctions, first described by Kraepelin [1] and Bleuler [2], have been observed in all phases of schizophrenia, from the premorbid period, through the ultra-high-risk state [3,4], the first episode of psychosis [5,6], and continuing into the chronic course of the disease [7,8]. The deficits are similar in different phases of disease and affect attention, working memory, executive functions, memory, language, and speed of processing. Despite the widespread presence of cognitive dysfunctions in individuals with schizophrenia spectrum disorders, there is only limited information on their underlying neurobiological mechanisms [9,10,11]. While many hypotheses have been posited regarding their etiology, it has recently been suggested, following Wernicke’s ideas, that cognitive dysfunctions are due to disconnection across the brain [12,13]. It is believed that the disruptions of the corpus callosum (CC)—the largest commissural fiber bundle in the brain connecting both hemispheres—could be responsible for some of the deficits [14].

The CC is one of the most studied white matter structures. As early as 1844, Arthur Wigan drew attention to the potential influence of this structure on mental disorders [15]. It is the largest white matter bundle in the central nervous system [16], consisting of 300 million fibers [17]. Due to its location at the bottom of the cerebral fissure, it plays an important role in the connection of the brain’s hemispheres, enabling distant brain structures on the left and right of the brain to communicate and coordinating cognitive, mental, and volitional processes [18]. Hofer and Frahm [17] segmented the corpus callosum into vertical subdivisions based on fiber tractography. They distinguished five regions crossed by fibers belonging to defined cortical areas, providing a more natural segmentation: Region 1—prefrontal areas; Region 2—premotor and supplementary motor areas; Region 3—primary motor areas; Region 4—primary sensory areas; and Region 5—parietal, temporal, and visual areas. Disturbances in the structure of this bundle and function are considered to be the biological basis of psychotic disorders [19]. However, the results in the literature are still inconclusive, especially regarding the segmentation of the CC and its relationship to schizophrenia [20,21].

Changes in the CC have been widely reported in patients with schizophrenia. Post-mortem studies have identified decreased density of astrocytes [22] and lower levels of neurofilaments [23]. Diffusion tensor imaging (DTI) made it possible to evaluate the properties of white matter in vivo and detect reduced CC integrity in patients with schizophrenia spectrum disorders, including first-episode psychosis and the ultra-high-risk state [24]. The meta-analysis of DTI studies on patients with chronic schizophrenia (CS) and patients with first-episode schizophrenia (FES) by Zhuo et al. [25] identified the genu and splenium of the CC as two areas with a reduced fractional anisotropy (FA) value when compared with HC. The ENIGMA Schizophrenia DTI Working Group (ENIGMA-SZ DTI) presented data showing significant differences in FA and mean diffusivity (MD) in the body and genu of the CC in patients with CS when compared to HC [26]. The team of Madigand et al. [27] confirmed differences between schizophrenia patients and HC in terms of microstructural alterations and revealed that patients with short and long illness duration did not differ from each other, while macrostructural changes were detected only for patients with long illness duration. Moreover, longitudinal studies have confirmed that after a baseline scan, patients with schizophrenia exhibit a more pronounced decline in the absolute size of the CC than healthy controls (HC [28,29]).

Despite the results indicating reduced FA in patients with FES, changes in integrity may already be present before the onset of the first episode [30]. Changes in FA in the CC depend on the age of the patients suffering from schizophrenia, with adolescents having a greater reduction in FA in anterior CC areas and adults having greater FA reduction in the posterior area [31,32]. It seems that, initially, the decrease in the FA value is located in the anterior part of the CC, and further areas of the CC are affected as the disease progresses [33,34]. Kong et al. [35] showed significantly decreased FA in the genu of the CC in FES when compared to CS, and only patients with CS significantly differed from HC. Similarly, Friedman et al. [36] did not find a significant difference in FA in the genu or splenium of the CC between FES patients and young HC; however, CS and older HC also did not differ significantly in this study. Likewise, Collinson et al. [37] did not find significant differences between clinical groups in FA in five different parts of the CC (anterior, mid-anterior, central, mid-posterior, and posterior).

In this study, we analyzed the three cognitive dimensions which are most investigated in the context of schizophrenia spectrum disorders and have the greatest influence on patients’ everyday functioning [38] (see above for meta-analysis [3,4,5,6,7,8]): working memory, which consists of the phonological loop (responsible for the temporary storage and maintenance of verbal material), the visuospatial sketchpad (responsible for visual and spatial information), and the central executive (in charge of controlling and regulating cognitive processes [39]); planning—the ability to identify and organize the steps and elements needed to achieve a goal [40]; and speed of processing, which can be operationalized as the number of correct responses generated during a task within a given amount of time or as the speed with which different cognitive operations can be performed [41,42].

To date, there has been little research on the differences between FES and CS. Most studies have found that patients with FES and CS have similar results on these three cognitive dimensions [43,44,45,46,47,48]. However, Wu et al. [49] found that FES patients had higher results for verbal working memory and planning than patients with CS. In contrast, Carolus et al. [50] found that patients with CS had higher results for planning and speed of processing than patients with FES. Although these impairments appear relatively stable during the course of the illness [51], there is also evidence of further widespread neurodegenerative progress [52] or cognitive decline in selected domains [53,54].

The relationship between abnormalities of the CC and cognitive functions has been less investigated in different stages of schizophrenia. Previous studies on CS demonstrated the relationship between FA in the different regions of the CC and the integration of information [55], planning [56], and speed of processing [57,58]. However, other reports did not confirm these findings regarding correlation with speed of processing [59,60,61,62]. There is also disagreement about the relationship between the integrity of the CC and cognitive functions in FES [63]. Some papers have found no correlations between FA and/or MD of the CC and working memory, planning, and speed of processing in FES patients [64,65,66,67,68]. Moreover, Yang et al. [69] did not confirm such relationships in never-treated FES.

Better understanding the brain connectivity underlying the cognitive dysfunctions in schizophrenia is important for developing biological and psychosocial intervention strategies to treat neurocognitive dysfunctions in schizophrenia spectrum disorders. The results of previous studies in this area remain contradictory and inconclusive. There is a paucity of reports examining the relationship between the integrity of the CC and different aspects of cognitive functioning in FES and CS simultaneously, and most studies did not analyze other DTI parameters, such as MD. Given the limitations of the above findings, to further clarify the relationship between FA and MD of the CC and working memory, planning, and speed of processing, the following study was designed with three aims. The first was to compare measures of the integrity of the CC (FA and MD) in patients with FES, patients with CS, and HC, controlled for age. The second aim was to compare the aforementioned cognitive abilities between the three studied groups, again with age being controlled for. The third aim was to estimate the relationship between the DTI measures and cognitive functioning, controlling for potential confounding factors in the three studied groups. Based on the available literature, we proposed three hypotheses. Firstly, there are differences between FES, CS, and HC in terms of the integrity of CC (FA and MD). We also hypothesized that there are differences between all groups in levels of cognitive performance. Our last hypothesis was that there is a relationship between DTI parameters and cognitive functions in all groups.

## 2. Materials and Methods

### 2.1. Participants

Eighteen patients with FES, 55 patients with CS, and 30 HC took part in this study. All participants were non-consanguineous and right-handed. The clinical groups were defined based on duration of illness; the FES group contained patients with very recent onset, while the CS group included patients with a minimum of ten years since onset. Properly licensed psychiatrists confirmed these diagnoses using a structured clinical interview based on the International Statistical Classification of Diseases and Related Health Problems (ICD-10 [70]) and Mini-International Neuropsychiatric Interview (MINI [71]). We recruited participants from among inpatients, day treatment patients, and outpatients at the Clinic of Psychiatry of the Pomeranian Medical University in Szczecin (Poland). The inclusion criteria required patients to be aged 18–40 for FES and 30–55 for CS (minimum 10 years of illness duration), comprehension of the test procedure, and to be in a stable clinical state. Due to the small size of the sample, we analyzed the results of one FES patient aged 41 and one CS patient aged 57 as an exceptional measure. Comorbid mental/neurological diseases, craniocerebral injuries, severe somatic diseases (e.g., cancer), dementia, and substance use disorders constituted exclusion criteria. The MRI and neuropsychological assessment were administered after one month of clinical stabilization of acute psychotic symptoms in FES in order to enhance cooperation and avoid the acute effects of psychotic symptoms [43]. We also excluded patients with CS who exhibited clear symptoms of disorganization (measured by “Conceptual disorganization” and “Unusual thought content” from the Positive and Negative Syndrome Scale; PANSS).

The HC group was comprised of persons without mental or neurological disorders who were matched in terms of sex and years of education. Students and employees of local universities disseminated information about the recruitment of HCs. Inclusion and exclusion criteria for this group were the same as the criteria for patients, with the exception of the clinical parameters pertaining to schizophrenia. No recruited individual was excluded based on the exclusion criteria. All participants gave written consent to participate in the study. The study protocol was approved by the local bioethics committee.

### 2.2. Neuropsychological Assessments

Four tasks from the Polish version of the Measurement and Treatment Research to Improve Cognition in Schizophrenia (MATRICS [72,73]) were included in this investigation.

For assessing visuospatial memory, the Spatial Span Subtest from the Wechsler Memory Scale was used. This task requires the participant to remember the locations of a series of blocks indicated by the person administering the test, forwards and backwards, respectively. Based on data collected and reviewed by Cornoldi and Mammarella [74], we measured visuospatial short-term memory using scores on the forwards version (WMS-SSSF), and visuospatial working memory using scores on the backwards version (WMS-SSSB) of this task.

For assessing verbal working memory, the Letter Number Span Test (LNST) was used. In this task, the participant must mentally reorder a list of letters and numbers that is presented orally and then to repeat it back. Based on the recommendations of Nuechterlein et al. [73], we analyzed the sum of correct answers of this task.

For assessing planning ability, we used the Mazes Subtest from the Neuropsychological Assessment Battery, which includes 7 mazes that gradually increase in difficulty. Each maze was distributed on a single sheet of paper and had to be completed using a pencil. Based on data collected and reviewed by Lace et al. [75], we measured planning independently of speed of processing using the sum of the mazes completed (NABMS), and planning dependent on speed of processing using the sum of points awarded based on the time taken to solve the mazes (NABMSP).

For assessing speed of visual processing, we used the Symbol Coding Subtest from the Brief Assessment of Cognition in Schizophrenia (BACSSCS), which requires subjects to write numbers corresponding to nonsense symbols as quickly as they can over a period of 90 s. Based on the recommendations of Nuechterlein et al. [73], we analyzed the sum of correct answers on this task.

### 2.3. Clinical Assessments

The Positive and Negative Syndrome Scale (PANSS [76]) was used to measure the severity of psychopathological symptoms in both clinical groups. Following the suggestions of Shafer and Dazzi [77], we distinguished five psychopathological dimensions: positive symptoms (items: P1 Delusions, P3 Hallucinatory behavior, P5 Grandiosity, P6 Suspiciousness and persecution, G9 Unusual thought content), negative symptoms (items: N1 Blunted affect, N2 Emotional withdrawal, N3 Poor rapport, N4 Passive apathetic social withdrawal, N6 Lack of spontaneity, G7 Motor retardation, G16 Active social avoidance), disorganization (items: P2 Conceptual disorganization, N5 Difficulty in abstract thinking, N7 Stereotyped thinking, G5 Mannerisms/posturing, G10 Disorientation, G11 Poor attention, G13 Disturbance of volition, G15 Preoccupation), affect (items: G1 Somatic concern, G2 Anxiety, G3 Guilt feelings, G4 Tension, G6 Depression), and resistance (items: P4 Excitement, P7 Hostility, G8 Uncooperativeness, G14 Poor impulse control). The Mini-International Neuropsychiatric Interview (MINI [71]) was used as a short structured diagnostic interview for psychiatric disorders. The severity of schizophrenia and its impact on functioning were assessed using the Global Assessment of Functioning (GAF [78]).

### 2.4. Acquisition of DTI

We obtained DTI data using a 3-Tesla scanner (General Electric Signa HDxt). The pulse sequence was single-shot, diffusion-weighted, echo planar acquisition: repetition time/echo time = 11,675 s/82.80 ms; numbers of excitation (NEX) = 2; acquisition time = 10 min, 19 s; matrix = 96 × 96; field of view = 240 mm × 240 mm; slice thickness = 3 mm; slice gap = 0.50; *b* value = 1000 s/mm^2^; diffusion gradient directions = 25.

### 2.5. Image Processing and Quality

**Figure 1:**
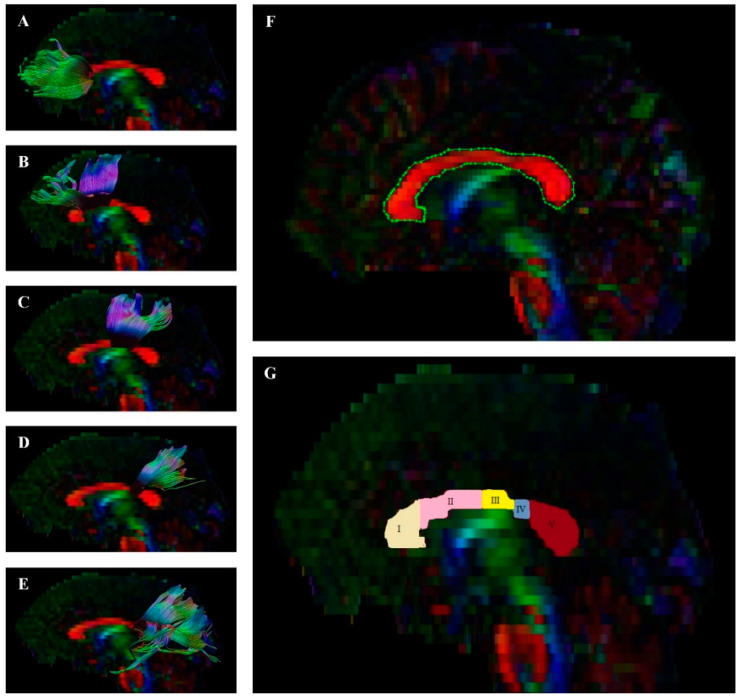
Diffusion tensor tractography of the corpus callosum (CC) with fractional anisotropy color maps (mid-sagittal plane). Green, red, and blue colors represent fibers running along the axis (anterior-posterior, left-right, and superior-inferior, respectively). Five single projections with ROIs on a sagittal plane ((**A**) = Region 1, (**B**) = Region 2, (**C**) = Region 3, (**D**) = Region 4, (**E**) = Region 5), (**F**) = whole region of CC, and (**G**) = all regions of CC (based on Hofer and Frahm’s [16] segmentation: I = Region 1, II = Region 2, III = Region 3, IV = Region 4, V = Region 5). The methodology and segmentation scheme are discussed in our previous publication [80].

First, preprocessing, quality control, and fiber tract visualization were performed with the ExploreDTI tool [79]. DICOM files were converted to the *.nii format, which is compatible with this program. Then, the sides of the converted images were checked to verify they matched the originals. Next, data were corrected for signal drift and effects due to motion and eddy current; artifacts, such as Gibbs ringing, were removed. Based on this data, whole-brain tractography was performed. To visualize the whole corpus callosum, a single region of interest (ROI) on the sagittal plane was used. Then, the CC was divided into 5 single ROIs on the sagittal plane using the method proposed by Hofer and Frahm [16] as presented on Figure 1. Following this, parts of tracts that were not anatomically involved were excluded as “ROInot” regions. FA and MD of the fiber tract was calculated automatically by the ExploreDTI Descriptive Statistics function.

### 2.6. Procedure

The research procedure comprised three parts: a psychiatric examination by one of four psychiatrists who carried out a structured interview (e.g., PANSS, MINI, GAF), neuropsychological assessment, carried out by one of five trained psychologists, and standardized MRI scanning. All participants were examined with the same neuropsychological tools. The standard instructions preceded the administration of each tool. The assessments were made between 8 a.m. and 3 p.m. and MRI scanning was done up to seven days after the neuropsychological assessment.

### 2.7. Statistical Analysis

Statistical analysis of the results was done using IBM SPSS 26 (IBM Corp., Redmont, VA, USA). Continuous variables were presented as means (*M*) and standard deviations (*SD*). The normality of distributions was tested with the Shapiro–Wilk test as well as skewness and kurtosis values. Differences between the two clinical groups were examined with Student’s *t* test and differences between the three groups were examined with one way analysis of variance (ANOVA). For significant differences in DTI measures and/or cognitive functions between three groups, analysis of covariance (ANCOVA) was performed to control the effect of age. Moreover, in the case of significant differences in cognitive functions between the two clinical groups, we conducted an ANCOVA to control the effects of age, duration of illness, exacerbations (defined as the number of acute exacerbations of psychotic symptoms in the course of the illness), and positive symptoms. Games-Howell (for ANOVA) and Bonferroni (for ANCOVA) post hoc tests were used for comparisons between groups. Cohen’s *d* or ɳ^2^ [81] was used to determine the magnitude of effect sizes for ANOVAs and ANCOVAs, respectively. Finally, in order to assess the relationships between DTI measures and cognitive functions in all groups, Pearson’s *r* correlation coefficient was estimated. Multivariate stepwise regression was performed for bivariate correlations in clinical groups that appeared to be significant with adjustment, controlling for the effects of clinical variables (i.e., duration of illness, exacerbations, chlorpromazine equivalent, positive symptoms, negative symptoms, disorganization, affect, and resistance). Separate single stepwise linear regression was performed for bivariate correlations in the control group that appeared to be significant. The variance inflation factor (VIF: >10) was calculated to assess multicollinearity. Holm-Bonferroni *p*-value correction was used for all statistical analyses with multiple comparisons and correlations. The alpha criterion level was set at 0.05 for all statistical analyses, but we also interpreted the effect of the difference as a statistical trend on level (0.05 < *p* < 0.1), which is recommended in the literature of biomedical sciences [82].

## 3. Results

### 3.1. Participant Characteristics

Demographic and clinical characteristics are presented in Table 1 (see Supplementary Table S1 for more details). There were no significant group differences in years of education or sex. However, there was a significant difference between groups in age (*F*_(2,100)_ = 14.58; *p* < 0.001; ɳ^2^ = 0.23). Post hoc analyses showed that patients with FES were younger (*p* < 0.001 and *p* = 0.001) than patients with CS and HC.

The clinical groups did not significantly differ (after Holm-Bonferroni *p*-value correction) in antipsychotic medications, chlorpromazine equivalent, global functioning measured by GAF, negative symptoms, disorganization, affect, or resistance measured by PANSS. However, patients with FES had shorter duration of illness (*p* < 0.001), fewer exacerbations (*p* < 0.001), and greater severity of positive symptoms (*p* = 0.008) than patients with CS.

### 3.2. Differences in DTI Measures

**Figure 2:**
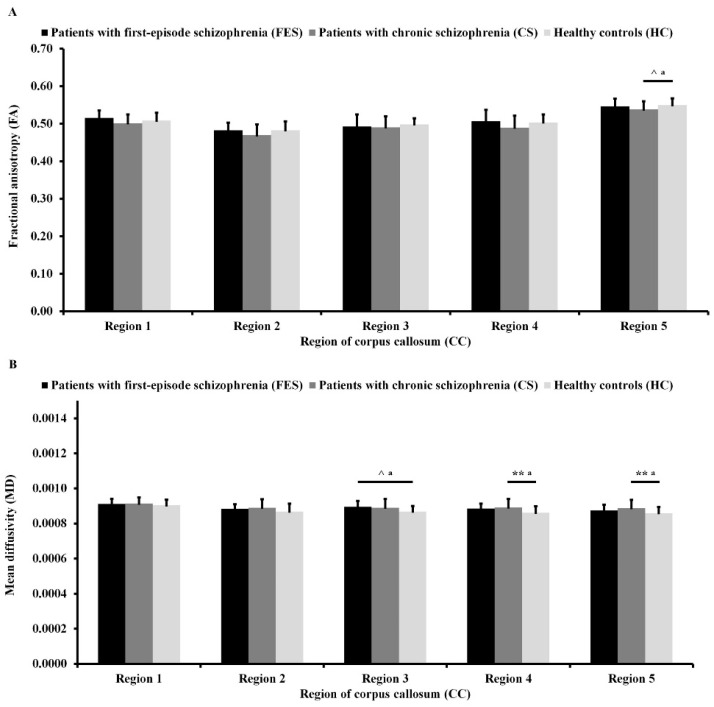
Fractional anisotropy (**A**) and mean diffusivity (**B**) of different regions of the corpus callosum for all participants. ^a^ Significant difference after co-varying age.^^^ *p* < 0.1. ** *p* < 0.01.

As can be seen in Figure 2A, there were trends toward statistical significance of difference in FA in Region 4 (*F*_(2,99)_ = 2.65; *p* = 0.076; ɳ^2^ = 0.05) and Region 5 (*F*_(2,99)_ = 2.65; *p* = 0.076; ɳ^2^ = 0.05) between the three groups after adjusting for age. Post hoc analyses showed that patients with CS had lower FA in Region 5 of CC (*p* = 0.077) than HC.

As can be seen in Figure 2B, there were significant differences in MD in Region 3 of the CC (*F*_(2,99)_ = 3.53; *p* = 0.033; ɳ^2^ = 0.07), Region 4 of the CC (*F*_(2,99)_ = 5.08; *p* = 0.008; ɳ^2^ = 0.09), and Region 5 of the CC (*F*_(2,99)_ = 4.83; *p* = 0.010; ɳ^2^ = 0.09) between the three groups after adjusting for age. Post hoc analyses showed that patients with FES had higher MD in Region 3 of the CC (*p* = 0.063) than HC on the level of trend toward statistical significance. Moreover, patients with CS had higher MD in Region 4 of the CC (*p* = 0.006) and Region 5 of the CC (*p* = 0.008) than HC.

### 3.3. Differences in Cognitive Functions

**Figure 3:**
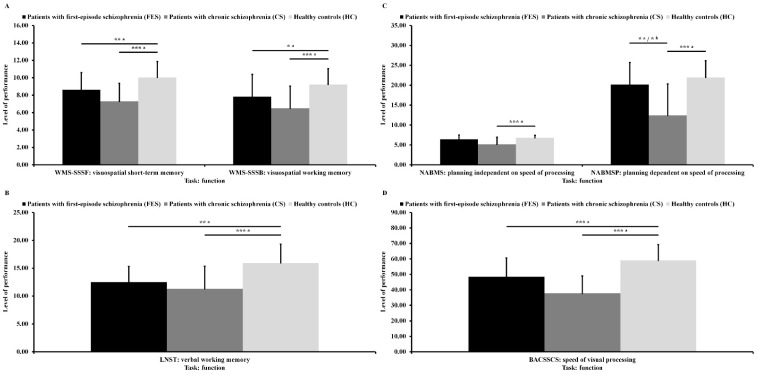
Performance of visuospatial memory (**A**), verbal working memory (**B**), planning ability (**C**), and speed of visual processing (**D**) for all participants. ^a^ Significant difference after co-varying age. ^b^ Significant difference after co-varying age, duration of illness, exacerbation, and positive symptoms. * *p* < 0.05. ** *p* < 0.01. *** *p* < 0.001.

As can be seen in Figure 3A, there were significant differences in visuospatial short-term memory (*F*_(2,99)_ = 18.25; *p* < 0.001; ɳ^2^ = 0.27) and in visuospatial working memory (*F*_(2,99)_ = 12.38; *p* < 0.001; ɳ^2^ = 0.20) between the three groups after adjusting for age. Post hoc analyses showed that patients with FES and patients with CS had lower results in the first variable (*p* = 0.003 and *p* < 0.001) and in the second variable (*p* = 0.049 and *p* < 0.001) than HC.

As can be seen in Figure 3B, there was a significant difference in verbal working memory (*F*_(2,99)_ = 15.28; *p* < 0.001; ɳ^2^ = 0.24) between the three groups after adjusting for age. Post hoc analyses showed that patients with FES and patients with CS had lower results in this variable (*p* = 0.004 and *p* < 0.001) than HC.

As can be seen in Figure 3C, there were significant differences in planning independent of speed of processing (*F*_(2,99)_ = 12.45; *p* < 0.001; ɳ^2^ = 0.20) and in planning dependent on speed of processing (*F*_(2,99)_ = 19.41; *p* < 0.001; ɳ^2^ = 0.28) between the three groups after adjusting for age. Post hoc analyses showed that patients with CS had lower results for first variable (*p* < 0.001) than HC. Moreover, patients with CS had lower results for the second variable (*p* < 0.045 and *p* < 0.001) than patients with FES as well as HC. Differences between clinical groups in planning dependent on speed of processing remained significant (*F*_(1,67)_ = 4.93; *p* = 0.030; ɳ^2^ = 0.07) after co-varying age, duration of illness, exacerbations, and positive symptoms.

As can be seen in Figure 3D, there was a significant difference in speed of visual processing (*F*_(2,99)_ = 34.85; *p* < 0.001; ɳ^2^ = 0.41) between the three groups after adjusting for age. Post hoc analyses showed that patients with FES and patients with CS had lower results in this variable (*p* < 0.001 and *p* < 0.001) than HC.

### 3.4. Relationship between DTI Measures and Cognitive Functions

**Figure 4:**
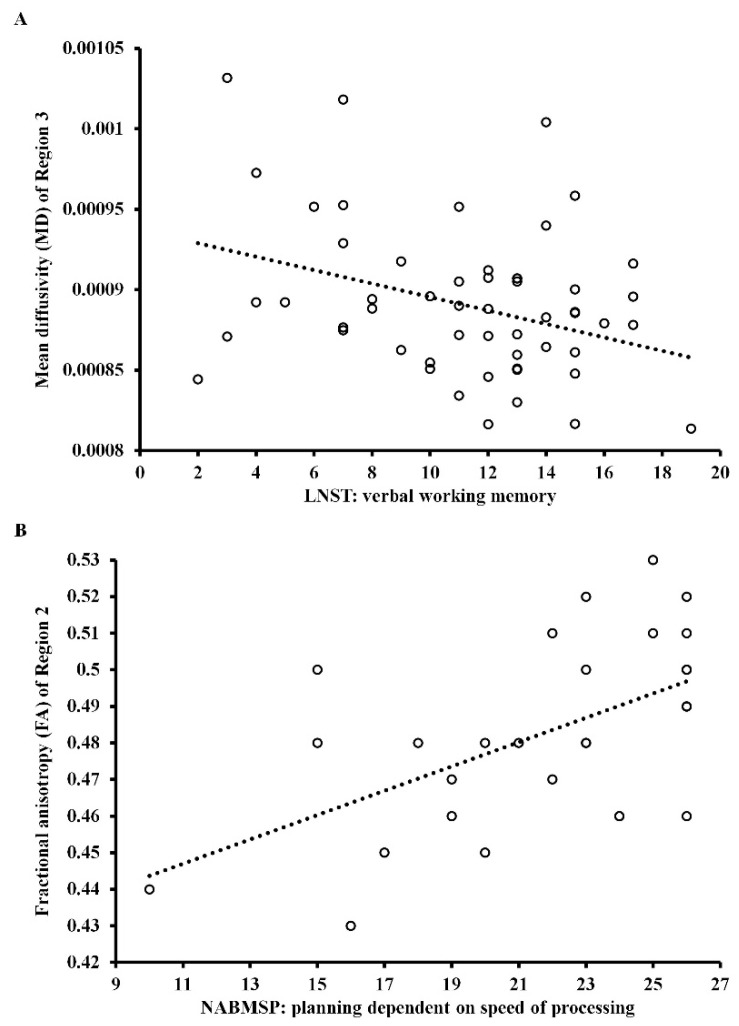
Scattergram for the relationship between: (**A**) mean diffusivity and verbal working memory in patients with chronic schizophrenia and (**B**) fractional anisotropy and planning dependent on speed of processing in healthy controls.

Figure 4A shows the significant correlation of MD in Region 3 of the CC with verbal working memory in patients with CS (*r* = −0.34; *p* = 0.050), and Figure 4B shows a significant correlation of FA in Region 2 of the CC with planning dependent on the speed of processing in healthy controls (*r* = 0.53; *p* = 0.015). Both correlation coefficients have been corrected (Holm-Bonferroni *p*-value correction). In patients with CS, the level of MD in Region 3 of the CC and negative symptoms were significant predictors of verbal working memory (β = −0.35; *t* = −2.86; *p* = 0.006 and β = −0.35; *t* = −2.88; *p* = 0.006), predicting about 21% of the variance (the model was well suited to the analyzed data, *F* = 8.15; *p* = 0.001). The other independent variables introduced into the model were statistically excluded.

In HC, the level of FA in Region 2 of the CC was a significant predictor of planning dependent on speed of processing (β = 0.53; *t* = 3.29; *p* = 0.003), predicting about 25% of the variance (the model was well suited to the analyzed data, *F* = 10.81; *p* = 0.003). All correlations between DTI measures and cognitive functions in three studied groups are provided in Supplementary Table S2.

## 4. Discussion

This study aimed to compare the structural integrity of different parts of the CC between patients with FES, patients with CS, and matched HC, controlling for effect of age. The results revealed lower FA in Region 5 (statistical trend) and higher MD in Regions 4 and 5 in patients with CS than HC, which confirms previous findings [25] but is inconsistent with results obtained by the ENIGMA-SZ DTI consortium [26]. Moreover, patients with FES had higher MD in Region 3 than HC (statistical trend), which is in line with previous results from Long et al. [65]. We did not find differences between both clinical groups in terms of FA or MD in any region of the CC, which corresponds with the results of Collinson et al. [37]. However, this lack of differences is not consistent with the findings of Kong et al. [35]. One explanation for this discrepancy might be the inclusion of patients of different ages and durations of illness. To avoid these problems, we controlled the effect of age between groups using it as a co-variate, similar to Collinson et al. [37]. Nevertheless, structural abnormalities in CS may be due to disturbances in myelination processes [83,84]. In general, disturbances in white matter integrity appear to be less consistent in patients with FES than in those with CS, suggesting that in the early stages of schizophrenia, there may be little or no disruption to the functional integrity of the corpus callosum, at least in some patients [85].

The second aim was to compare cognitive abilities between the three studied groups while controlling for age. We found that patients from both clinical groups had disturbances in both visuospatial and verbal working memory as well as in speed of processing. These results are in line with the findings of most previous studies that used the same tests from the MATRICS battery [43,45,47,49,50]. However, our results are inconsistent with other studies that showed no group differences in working memory or speed of processing [44,46,48]. We found that patients with CS displayed impairments in both aspects of planning in comparison to HC and had greater difficulties with planning than patients with FES, independently of speed of processing. It is worth highlighting that in most previous studies, patients from both clinical groups had lower results than HC, but were not different in terms of planning; however, this ability was measured based on only one indicator that depended on the speed of processing. Whereas Wu et al. [49] showed that patients with CS had greater difficulties than patients with FES, Carolus et al. [50] found the opposite. We can explain these discrepancies by observing that we analyzed raw scores on all tests while controlling age as a co-variate, whereas most other authors analyzed normalized scores for seven cognitive dimensions, not tests separately and not always controlling demographic variables. Moreover, when we compared results for both clinical groups, we controlled the effects of age, duration of illness, exacerbations, and positive symptoms.

The third aim of this study was to estimate the relationships between DTI measures and cognitive functions, controlling for potential confounders in the three studied groups. We found a correlation between MD in Region 3 and verbal working memory in patients with CS. These findings are consistent with the results of Kochunov et al. [86], but not with the results from other studies [58,69]. The relationship between this region of the CC and verbal working memory may be explained in terms of disturbances in communication between motor areas. These brain regions are parts of a pericentral network involved in motor and somatosensory processing [87]. According to Marvel et al. [88], brain areas involved in working memory are organized along a motor-cognitive gradient, where neurons from the motor and cognitive networks interact with each other. There were no significant relationships between DTI measures and different aspects of cognitive functions in patients with FES, which is consistent with some previous findings [64,65,66,67,68]. In addition, we found a relationship between the integrity of Region 2 of the CC and planning dependent on speed of processing in HC. These findings are partly consistent with previous studies [89,90]. This relationship may be due to synchronization within the lateral frontoparietal network that is involved in attention processing [87].

Taken together, these results are generally in accordance with the neurodevelopmental model of schizophrenia, rather than with the neurodegenerative approach, which accommodates tiered interaction between brain pathology, genetics, environmental factors, and gene-environmental interactions [91,92]. Expanding on the neurodevelopmental model, the disconnection brain theory [12,13] may explain the disruptions in the CC, cognitive impairments, and their relationship at different stages of the schizophrenia spectrum. On the one hand, the presence of CC integrity disturbances in CS and the lack thereof in FES may suggest progressive changes in white matter. On the other hand, impairments in cognitive dimensions in both clinical groups suggest stabilization of these dysfunctions. However, brain development is an ongoing process that occurs throughout life, and changes in its communication in schizophrenia are more subtle than pronounced; therefore, it is not fully possible to draw final conclusions at this stage of research [93].

The results of the present study must be seen in the context of its limitations. First, the small sample of patients with FES limits generalizations for this stage of the illness, especially for the tractography analyses. Findings for FES may be viewed as preliminary but potentially useful, as they suggest directions for future research. Second, participants were evaluated by multiple research team members, which could be a source of potential differences in data collection. To minimize this risk, all team members were trained and followed the same research protocol. Third, while tractography is thought to be a powerful tool for studying white matter integrity in schizophrenia and other conditions, it has an important limitation related to the existence of crossing fibers that influence FA that could have contributed to the present findings. The bending, merging, and crossing of fibers in some voxels, together with low spatial resolution at 3T, could lead to inexact measures of FA [94]. Fourth, as this was a cross-sectional study, we did not conduct longitudinal analysis. Future research, specifically longitudinal studies, should investigate the influence of illness duration and medication on the relationship between white matter integrity of the CC and cognitive functions for the whole schizophrenia spectrum, from ultra-high-risk, through first-episode, to chronic schizophrenia. Fifth, we did not calculate other DTI measures, such as axial diffusivity (AD) or radial diffusivity (RD). Sixth, we did not control premorbid intellectual functioning between groups. Intellectual functioning can be associated with working memory, planning, and speed of processing, but previous results are inconclusive in this regard [95,96,97]. Finally, the ecological validity of the applied diagnostic methods was low [98], making it difficult to draw conclusions about patients’ cognitive performance in everyday life situations. Future research should investigate cognitive dimensions using more ecological tools.

## 5. Conclusions

In conclusion, our study found that patients with CS showed lower FA (statistical trend) and higher MD in the CC than HC, while patients with FES had higher MD in the CC (statistical trend) compared to HC. Further research is needed to verify the results of this study. Our results suggest that patients in both clinical groups perform worse on working memory and speed of processing tasks than HC. Patients with CS had lower results than patients with FES only for planning dependent on speed of processing. This study provides evidence that integration of the CC in patients with CS might account for impairments in planning ability. In the future, multimodal studies that integrate structural (white and gray matter) and functional (resting-state and task-based) imaging are necessary to understand further the neuro-pathophysiological basis of cognitive functions in schizophrenia spectrum disorders and identify the possible underlying alterations in neural networks.

## Figures and Tables

**Table 1:** Demographic and clinical characteristics of all participants.

	Patients with First-Episode Schizophrenia (FES) (*n* = 18)	Patients with Chronic Schizophrenia (CS) (*n* = 55)	HealthyControls (HC) (*n* = 30)	*F*/χ^2^/*t*
Age: *M* (*SD*)	28.39 (6.98)	39.09 (6.92)	37.30 (8.24)	14.58 *** ^a^
Years of education: *M* (*SD*)	13.28 (3.16)	13.24 (2.61)	14.53 (2.60)	2.40 ^a^
Sex: female/male	13/5	26/29	16/14	3.39 ^b^
Antipsychotic medications:				
Atypical: *n* (%)	14 (77.78)	37 (67.27)	-	2.55 ^b^
Atypical and typical: *n* (%)	2 (11.12)	15 (27.27)	-	
Typical: *n* (%)	1 (5.55)	2 (3.64)	-	
No medications: *n* (%)	1 (5.55)	1 (1.82)	-	
Chlorpromazine equivalent (mg): *M* (*SD*)	470.17 (331.53)	644.96 (301.72)	-	−2.08 ^c^
Duration of illness: *M* (*SD*)	0.44 (0.32)	14.73 (5.47)	-	−19.27 *** ^c^
Exacerbation: *M* (*SD*)	1.11 (0.32)	6.11 (3.89)	-	−9.43 *** ^c^
Global functioning in GAF: *M* (*SD*)	60.89 (14.73)	58.51 (13.80)	-	0.63 ^c^
Psychopathological symptoms in PANSS:				
Positive symptoms: *M* (*SD*)	11.61 (3.73)	7.85 (3.83)	-	3.64 ** ^c^
Negative symptoms: *M* (*SD*)	16.89 (5.96)	16.42 (6.30)	-	0.28 ^c^
Disorganization: *M* (*SD*)	13.78 (4.43)	11.64 (3.74)	-	2.01 ^c^
Affect: *M* (*SD*)	10.67 (4.26)	9.36 (3.63)	-	1.27 ^c^
Resistance: *M* (*SD*)	5.72 (2.16)	4.78 (2.23)	-	1.57 ^c^

GAF = Global Assessment of Functioning; PANSS = Positive and Negative Syndrome Scale. ^a^ One-way analysis of variance *F* test. ^b^ Chi-squared test. ^c^ Student’s *t* test. ** *p* < 0.01. *** *p* < 0.001.

## Data Availability

Materials of the study reported here are available from the corresponding author on reasonable request.

## Supplementary Materials

The following are available online at https://www.mdpi.com/article/10.3390/jcm10143158/s1,Table S1. Demographic and clinical characteristics of all participants, Table S2. Relationship between DTI measures of the corpus callosum (CC) and cognitive functions in all participants (Pearson’s *r* correlation coefficient).
