# Supplementary material for: Relationship of Corpus Callosum Integrity with Working Memory, Planning, and Speed of Processing in Patients with First-Episode and Chronic Schizophrenia
Source: J Clin Med. 2021 Jul 17;10(14):3158. doi: 10.3390/jcm10143158 (PMC8304050; doi:10.3390/jcm10143158)
Supplement: Supplementary file 1 [file jcm-10-03158-s001.zip › jcm-1293845-supplementary.pdf]

Supplementary Materials

Supplementary Table S1. Demographic and clinical characteristics of all participants.

	Patients with first-episode schizophrenia (FES) (*n* = 18)	Patients with chronic schizophrenia (CS) (*n* = 55)	Healthy controls (HC) (*n* = 30)	F / χ^2 / t	*p* uncorrected	*p* * corrected
Age: *M* (*SD*) / Min-Max	28.39 (6.98) / 19-41	39.09 (6.92) / 25-57	37.30 (8.24) / 22-48	14.58 [a]	0.000	-
Years of education: *M* (*SD*) / Min-Max	13.28 (3.16) / 6-17	13.24 (2.61) / 8-18	14.53 (2.60) / 11-19	2.40 [a]	0.096	-
Sex: female / male	13 / 5	26 / 29	16 / 14	3.39 [b]	0.183	-
Antipsychotic medications:						
Atypical: *n* (%)	14 (77.78)	37 (67.27)	-			
Atypical and typical: *n* (%)	2 (11.12)	15 (27.27)	-	2.55 [b]	0.467	-
Typical: *n* (%)	1 (5.55)	2 (3.64)	-			
No medications: *n* (%)	1 (5.55)	1 (1.82)	-			
Chlorpromazine equivalent (mg): *M* (*SD*) / Min-Max	470.17 (331.53) / 0-1500	644.96 (301.72) / 0-1500	-	-2.08 [c]	0.041	0.246
Duration of illness: *M* (*SD*) / Min-Max	0.44 (0.32) / 0.8-1	14.73 (5.47) / 10-27	-	-19.27 [c]	0.000	0.000
Exacerbation: *M* (*SD*) / Min-Max	1.11 (0.32) / 1-2	6.11 (3.89) / 1-24	-	-9.43 [c]	0.000	0.000
Global functioning in GAF: *M* (*SD*) / Min-Max	60.89 (14.73) / 30-88	58.51 (13.80) / 30-88	-	0.63 [c]	0.534	1.000
Psychopathological symptoms in PANSS:						
Positive symptoms: *M* (*SD*) / Min-Max	11.61 (3.73) / 5-18	7.85 (3.83) / 5-22	-	3.64 [c]	0.001	0.007
Negative symptoms: *M* (*SD*) / Min-Max	16.89 (5.96) / 7-25	16.42 (6.30) / 7-28	-	0.28 [c]	0.781	1.000
Disorganization: *M* (*SD*) / Min-Max	13.78 (4.43) / 8-23	11.64 (3.74) / 8-27	-	2.01 [c]	0.048	0.246
Affect: *M* (*SD*) / Min-Max	10.67 (4.26) / 5-21	9.36 (3.63) / 5-18	-	1.27 [c]	0.209	0.627
Resistance: *M* (*SD*) / Min-Max	5.72 (2.16) / 4-11	4.78 (2.23) / 4-19	-	1.57 [c]	0.122	0.488

GAF = Global Assessment of Functioning; PANSS = Positive and Negative Syndrome Scale.

[a] One-way analysis of variance *F* test. [b] Chi-squared test. [c] Student's *t* test.

* After Holm-Bonferroni *p*-value correction for multiple comparisons.

Supplementary Table S2. Relationship between DTI measures of the corpus callosum (CC) and cognitive functions in all participants (Pearson's *r* correlation coefficient).

Variable	Fractional anisotropy (FA)										Mean diffusivity (MD)									
	Region 1		Region 2		Region 3		Region 4		Region 5		Region 1		Region 2		Region 3		Region 4		Region 5	
	r	*p*	*r*	*p*	*r*	*p*	*r*	*p*	*r*	*p*	*r*	*p*	*r*	*p*	*r*	*p*	*r*	*p*	*r*	*p*
Patients with first-episode schizophrenia (FES) (*n* = 18)																				
WMS-SSSF: visuospatial short-term memory	0.06	0.816	0.14	0.591	0.41	0.087	0.01	0.954	-0.03	0.915	-0.14	0.592	0.08	0.752	-0.21	0.393	-0.08	0.765	-0.03	0.905
WMS-SSSB: visuospatial working memory	0.15	0.547	0.18	0.465	0.20	0.433	-0.01	0.975	0.05	0.838	0.23	0.363	0.36	0.140	0.17	0.504	0.42	0.084	0.37	0.133
LNST: verbal working memory	-0.10	0.689	-0.17	0.511	-0.13	0.605	0.11	0.662	-0.29	0.240	-0.22	0.376	-0.13	0.602	0.24	0.348	0.03	0.916	0.15	0.553
NABMS: planning independent on speed of processing	0.08	0.738	0.19	0.455	-0.06	0.825	0.06	0.823	-0.03	0.906	-0.15	0.544	-0.15	0.549	-0.14	0.568	-0.45	0.061	-0.24	0.344
NABMSP: planning dependent on speed of processing	0.27	0.273	0.40	0.103	0.20	0.423	0.10	0.707	0.04	0.874	-0.28	0.265	-0.35	0.155	-0.38	0.115	-0.53	0.023	-0.27	0.274
BACSSCS: speed of visual processing	-0.31	0.217	-0.29	0.245	-0.23	0.359	-0.30	0.224	-0.58	0.012	-0.13	0.611	0.09	0.711	0.15	0.550	0.05	0.852	0.19	0.441
Patients with chronic schizophrenia (CS)																				

(n = 55)																				
WMS-SSSF: visuospatial short-term memory	0.17	0.205	0.15	0.260	0.14	0.309	0.19	0.171	0.22	0.111	-0.19	0.173	-0.11	0.417	-0.16	0.241	-0.12	0.391	-0.10	0.477
WMS-SSSB: visuospatial working memory	0.05	0.704	0.11	0.426	0.06	0.688	0.07	0.624	0.13	0.355	-0.09	0.521	-0.03	0.806	-0.04	0.749	0.02	0.912	-0.11	0.411
LNST: verbal working memory	0.08	0.542	0.1	0.456	0.23	0.095	0.03	0.820	0.10	0.456	-0.21	0.131	-0.20	0.141	-0.34*	0.010	-0.14	0.294	-0.12	0.371
NABMS: planning independent on speed of processing	0.08	0.577	0.11	0.414	0.19	0.173	0.09	0.508	0.07	0.62	-0.14	0.318	-0.11	0.415	-0.17	0.221	-0.10	0.456	-0.04	0.744
NABMSP: planning dependent on speed of processing	0.07	0.607	0.09	0.504	0.15	0.271	0.04	0.749	0.05	0.712	-0.12	0.369	-0.07	0.591	-0.16	0.244	-0.19	0.160	-0.09	0.507
BACSSCS: speed of visual processing	-0.01	0.926	-0.06	0.650	0.11	0.441	0.00	0.987	0.20	0.145	-0.19	0.162	-0.07	0.607	-0.19	0.170	-0.17	0.208	-0.17	0.213
Healthy controls (HC) (n = 30)																				
WMS-SSSF: visuospatial short-term memory	0.28	0.137	0.34	0.064	0.06	0.770	0.15	0.441	0.20	0.300	0.17	0.365	-0.05	0.808	0.07	0.721	0.08	0.679	0.14	0.471
WMS-SSSB: visuospatial working memory	0.12	0.518	0.03	0.875	0.07	0.723	0.16	0.400	0.43	0.019	0.09	0.626	0.11	0.562	0.04	0.854	-0.04	0.848	-0.14	0.475
LNST: verbal working memory	0.14	0.468	0.32	0.084	0.05	0.808	0.11	0.550	-0.09	0.632	-0.02	0.929	-0.16	0.393	-0.03	0.887	0.09	0.638	0.08	0.678
NABMS: planning independent on speed of processing	0.00	0.994	0.11	0.560	0.09	0.647	0.24	0.194	0.13	0.495	0.10	0.602	-0.03	0.862	-0.20	0.286	-0.09	0.643	0.25	0.176
NABMSP: planning dependent on speed of processing	0.33	0.074	0.53*	0.003	-0.01	0.954	0.23	0.222	-0.08	0.663	0.02	0.920	-0.32	0.081	-0.20	0.297	0.00	0.996	0.31	0.092
BACSSCS: speed of visual processing	0.05	0.773	-0.06	0.736	0.09	0.629	-0.16	0.410	0.24	0.200	0.28	0.132	0.20	0.283	0.13	0.481	0.17	0.372	-0.09	0.646

* $p < 0.05$ (after Holm-Bonferroni p-value correction for multiple correlations).
